# Recurrent Atypical Fibroxanthoma in a 76-Year-Old Man Treated With Cryotherapy: A Case Report

**DOI:** 10.7759/cureus.102596

**Published:** 2026-01-29

**Authors:** Isaac Apichoto-Mata, Silvia Julieta García-Contreras, Brenda Garnica

**Affiliations:** 1 Internal Medicine, Facultad Mexicana de Medicina de la Universidad La Salle, Ciudad de México, MEX; 2 Dermatology, Universidad Nacional Autónoma de México, Ciudad de México, MEX

**Keywords:** afx, atypical fibroxanthoma, cryotherapy in afx, pleomorphic dermal sarcoma, recurrent afx

## Abstract

Atypical fibroxanthoma (AFX) is a rare cutaneous neoplasm that predominantly affects elderly individuals, typically arising on sun-exposed areas of the head and neck and exhibiting diverse clinical presentations. Histologically, it is composed of atypical spindle cells and is associated with intermediate-grade malignant potential. This case report describes a 76-year-old man diagnosed with recurrent AFX, initially presenting in 2022, with subsequent recurrences in 2023 and 2024 despite adequate excisional surgeries. The patient was ultimately treated with cryotherapy, achieving a 12-month recurrence-free period. Although surgical excision remains the gold standard, there are no universally accepted standardized therapies or guidelines for AFX management. Recent studies, however, have demonstrated the efficacy of non-surgical pharmacological treatments. This report highlights the diagnostic challenges and therapeutic complexities of recurrent AFX, particularly its propensity to mimic more aggressive malignancies.

## Introduction

Atypical fibroxanthoma (AFX) is a rare dermatologic neoplasm predominantly affecting elderly individuals in sun-exposed areas, particularly the head and neck. While it typically presents as a rapidly developing, ulcerated, and/or bleeding lesion, AFX can also manifest as non-pigmented macules and patches [1]. This varied presentation often complicates diagnosis, as it can be confused with other skin cancers and cutaneous lesions, including basal cell carcinoma, non-melanocytic melanoma, and squamous cell carcinoma. Diagnosis is typically confirmed through biopsy and histopathology, revealing positive expression of markers such as CD68, CD99, and CD10, while being negative for cytokeratin, desmin, CD34, S-100, and CKAE1/AE2 [2,3]. AFX originates from atypical spindle cells, a histological characteristic shared with pleomorphic dermal sarcoma (PDS), a type of soft tissue tumor. Some authors describe AFX as a superficial variant of PDS, a widely accepted view given PDS’s more aggressive clinical behavior involving vascular, perineural, or subcutaneous tissues. Others consider AFX a pre-stage lesion preceding the development of PDS; however, this assertion remains under discussion [2]. Both AFX and PDS are frequently linked to undifferentiated pleomorphic sarcoma, though their direct association remains unclear. Regarding treatment, studies indicate no significant difference in efficacy between wide local excision and Mohs’ micrographic surgery, making surgical procedures the accepted gold standard for AFX [4]. For recurrent cases, common therapies include radiotherapy and emerging management strategies with proapoptotic topical drugs [5,6]. Despite these advances, the high recurrence rate of AFX necessitates exploration of novel therapeutic approaches and a deeper understanding of its biological underpinnings [5]. This case report aims to further elucidate the clinical course, diagnostic considerations, and management challenges associated with recurrent AFX, thereby contributing to the limited body of literature on this often problematic neoplasm [4].

## Case presentation

**Figure 1:**
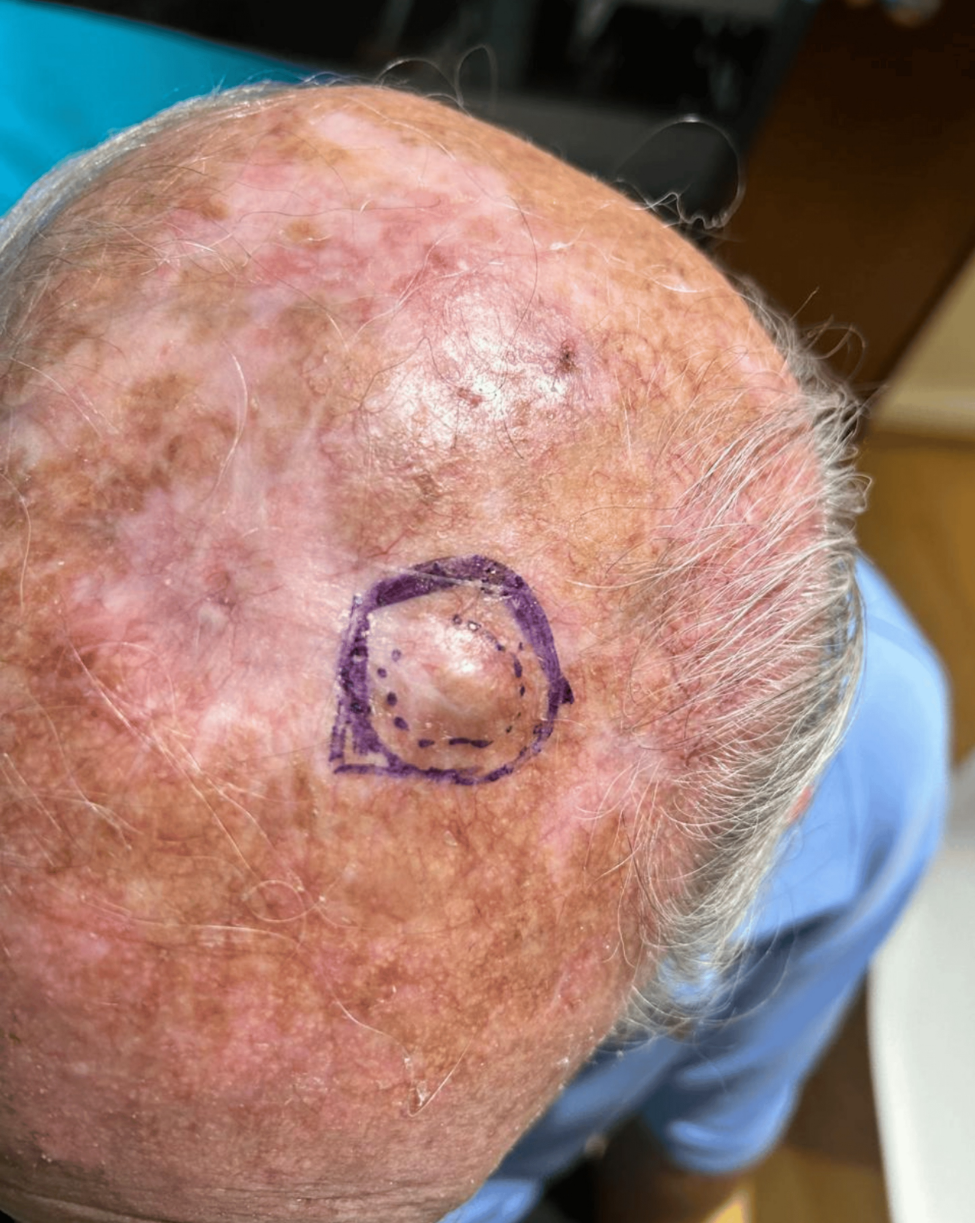
First presenting lesion: an elevated 2 × 2 cm nodule in the left parietal area.

**Figure 2:**
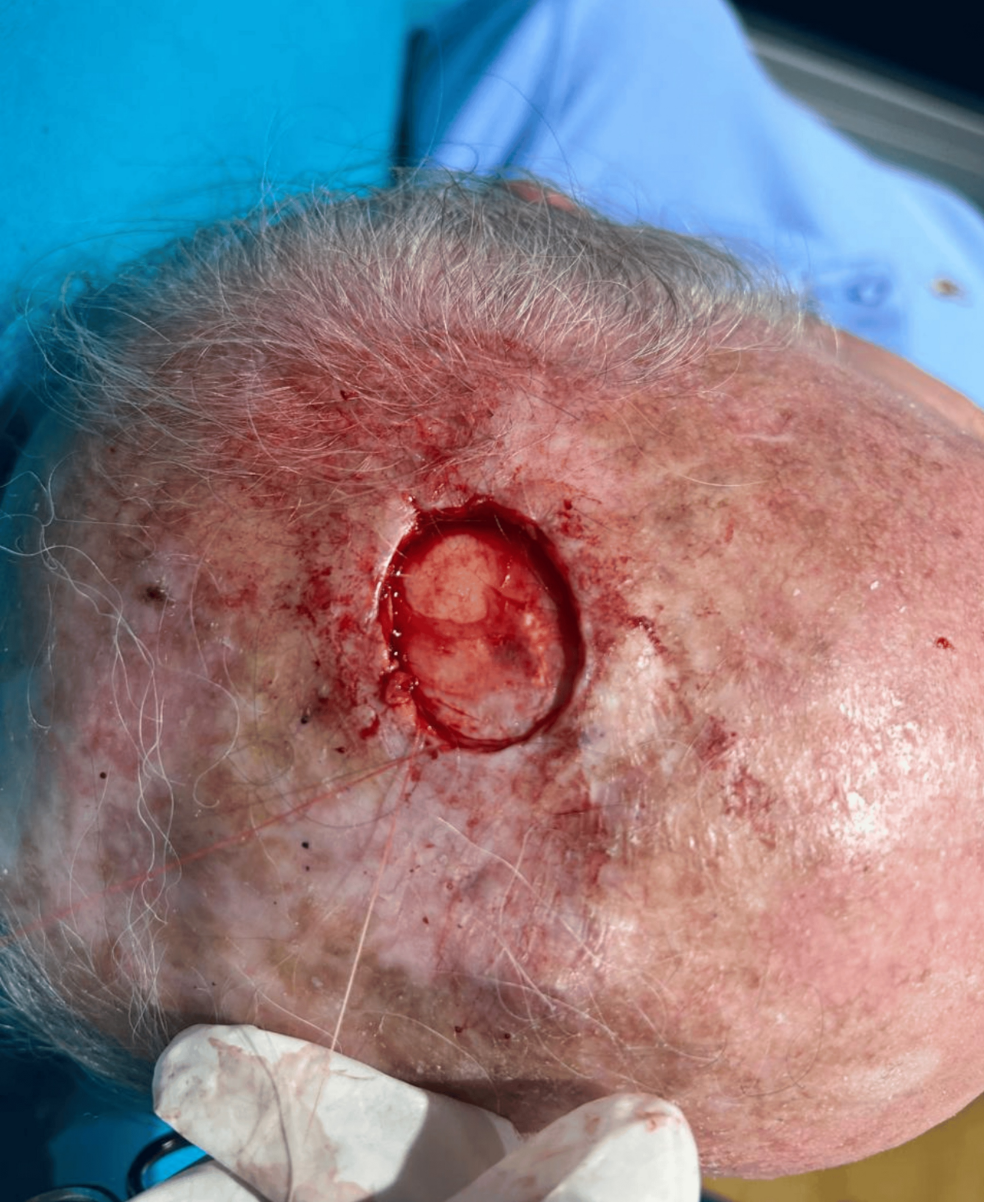
Excisional biopsy performed at initial presentation.

We report the case of a 76-year-old man with recurrent AFX. He initially presented to our clinic in 2022 with a verrucous lesion on the neck. His medical history was notable for lung cancer diagnosed one year earlier, which was in remission and asymptomatic. Physical examination identified a non-pigmented, elevated nodular lesion measuring 2 × 2 cm on the scalp (Figure 1). An excisional biopsy was performed for histopathological evaluation (Figure 2).

Pathological analysis revealed a low-grade, infiltrative fibrohistiocytic neoplasm with expansive margins, comprising fusiform cells with large, vesicular nuclei and minimal atypia, alongside ovoid cells exhibiting xanthomatous cytoplasm and nuclei with moderate-to-severe pleomorphism, including anaplastic features and mitotic activity. No necrosis or lymphovascular invasion was observed; however, the lesion extended into the reticular dermis with positive margins. Immunohistochemical staining was positive for CD68, CD10, and Ki67, and negative for S-100 and CKAE1/AE3, supporting the diagnosis and excluding other malignancies. A subsequent wide excision achieved clear margins, with 6 months of follow-up showing no recurrence.

**Figure 3:**
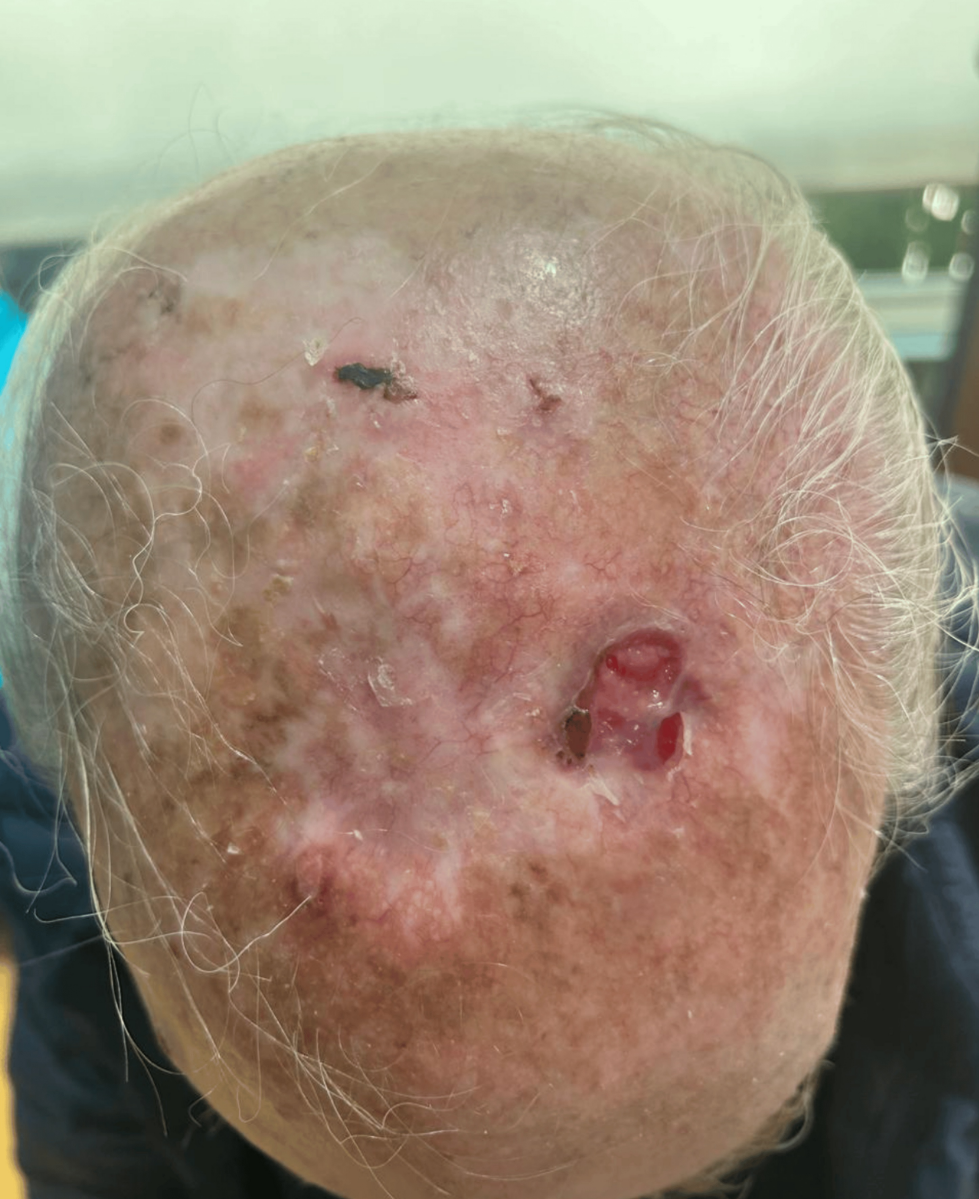
Recurrence two years later, presenting as an ulcerated, bleeding 1 × 3 cm lesion.

**Figure 4:**
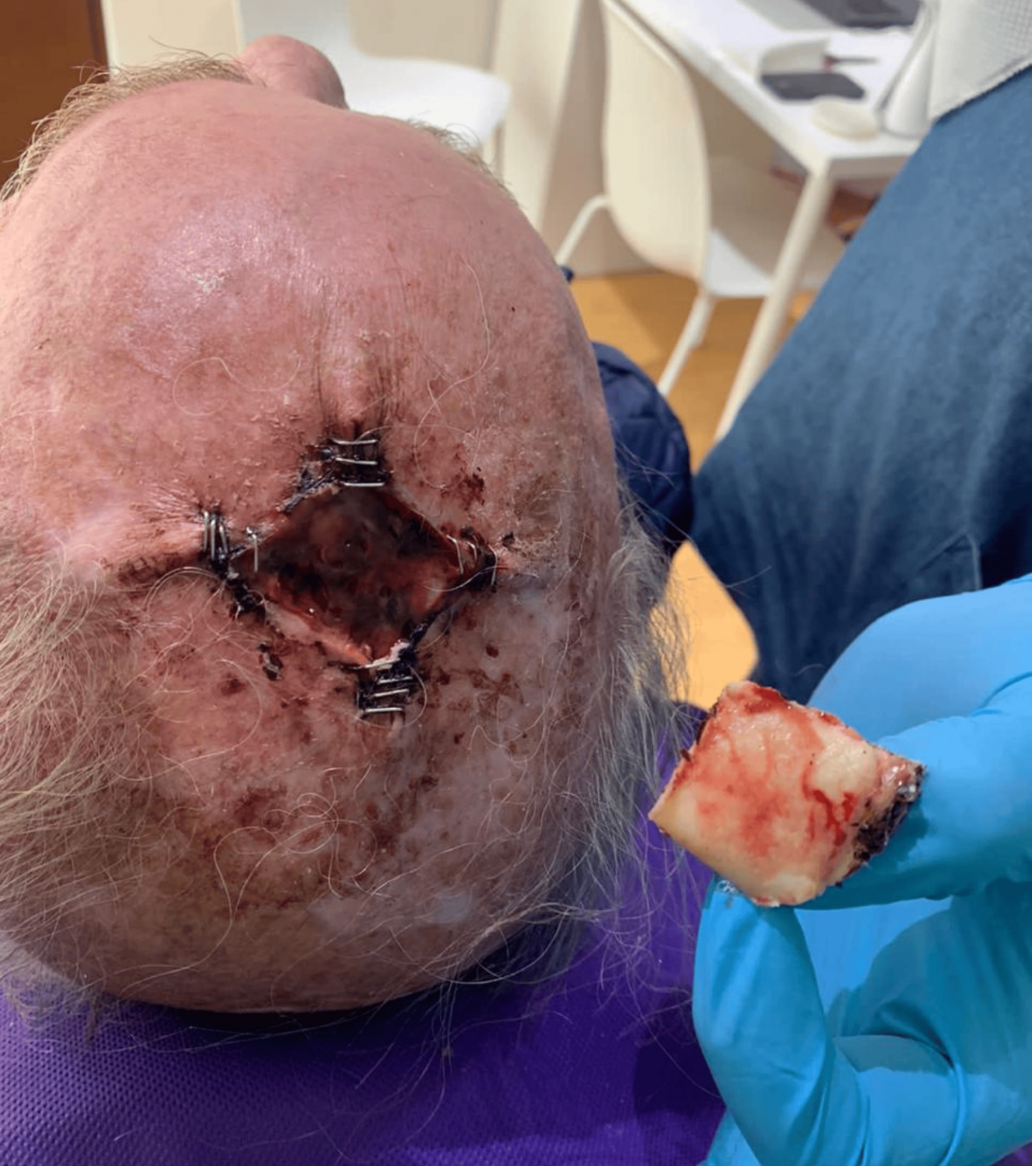
Wide excisional surgical procedure performed after the patient re-presented.

In January 2024, the patient re-presented with a 1 × 2 cm ulcerated, bleeding lesion on the scalp, accompanied by surrounding skin discoloration (Figure 3). Wide excision was performed (Figure 4), and histopathology confirmed recurrent AFX: a mesenchymal neoformation with a diffuse pattern, extending focally through the full thickness of the specimen, composed of fascicles and bundles of ovoid, spindled, and pleomorphic cells, some multinucleated, with pleomorphic nuclei, prominent nucleoli, and atypical mitotic figures.

Six months post-excision, a surveillance biopsy demonstrated incipient dermal fibrosis with abundant fibroblasts, collagen, perivascular inflammatory infiltrate, and granulation tissue, without residual fibrohistiocytic neoplasm, indicating successful clearance. However, six months later, a rapidly enlarging scalp nodule prompted further evaluation, suggestive of another recurrence. Mohs micrographic surgery was recommended but declined by the patient due to concerns over cosmetic and functional outcomes. Instead, cryotherapy was selected, despite counseling on its lower efficacy relative to surgery. The patient has since undergone 12 months of dermatologic surveillance without evidence of recurrence.

## Discussion

The clinical features observed in this case align with those generally described in the literature. Initially, the lesion presented as an elevated, hypochromic nodule measuring 2 × 2 cm, which was treated with two wide local excisional procedures to achieve clear margins. Two years later, the patient developed an ulcerated and bleeding lesion, histologically confirmed as a recurrence of AFX, and underwent excisional surgery. One year later, another recurrence emerged; this time, cryotherapy was chosen as a shared decision with the patient, who was reluctant to undergo further surgical intervention. Although this approach deviates from current management strategies for recurrent lesions, the patient has maintained a 12-month lesion-free period. Given the patient’s advanced age and history of recurrence, a more intensive follow-up regimen may be warranted to monitor for further local recurrences, particularly as elderly males face a higher risk [7,8].

Despite its typically indolent nature, AFX can display aggressive behavior, with recurrence rates varying from <2% to 8.0% following local excision and 4.6% after Mohs micrographic surgery [1,4]. This wide range in recurrence rates highlights the necessity of tailored management strategies, especially for atypical presentations or in situations where complete excision proves challenging [4]. Furthermore, the average time to recurrence for AFX is reported as 33.2 months, while the average time to metastasis is 39.6 months, underscoring the need for extended surveillance in such cases [4]. Although AFX typically exhibits a favorable clinical course, a meticulous examination of larger lesions for indicators such as necrosis, lymphovascular invasion, and subcutaneous extension is crucial to distinguish it from undifferentiated pleomorphic sarcoma, which carries a significantly higher risk of recurrence and metastasis [2,8-9].

Even with the low recurrence rates reported in the literature, it is imperative to study new approaches to standard treatment, especially considering the potential for re-recurrences and the challenges associated with managing recurrent lesions in elderly patients who may decline invasive procedures [4,10]. This case highlights the complexities involved in managing recurrent AFX, particularly when patients opt for less invasive treatments than recommended in guidelines [9,11]. Future research should therefore focus on developing targeted therapies or less invasive yet effective treatment modalities for recurrent AFX to accommodate patient preferences and improve long-term outcomes in vulnerable populations.

## Conclusions

AFX is considered a superficial sarcoma with an indolent course, characterized by low rates of recurrence and metastasis. Progression to pleomorphic dermal sarcoma is a rare complication that necessitates careful monitoring. Generally, it is considered a diagnosis of exclusion, as it shares many characteristics with other types of dermatologic malignancies. Therefore, accurate diagnosis relies on a combination of clinical assessment, histologic examination, and immunohistochemical profiling to differentiate it. Once recognized, AFX is typically managed surgically, with wide local excision or Mohs micrographic surgery being the preferred methods to achieve clear margins and minimize recurrence. Despite the efficacy of surgical intervention, a small percentage of cases, like the one presented, may experience recurrence, necessitating re-evaluation of treatment strategies and long-term surveillance. Given the invasive nature of the proposed treatments, it is well understood that patient compliance can be a significant factor, highlighting the need for a balanced approach between optimal oncologic outcomes and patient-centered care. Further research into novel therapeutic approaches for this condition is warranted to develop innovative treatment modalities.
